# Use of next generation sequencing data to develop a qPCR method for specific detection of EU-unauthorized genetically modified *Bacillus subtilis* overproducing riboflavin

**DOI:** 10.1186/s12896-015-0216-y

**Published:** 2015-11-11

**Authors:** Elodie Barbau-piednoir, Sigrid C. J. De Keersmaecker, Maud Delvoye, Céline Gau, Patrick Philipp, Nancy H. Roosens

**Affiliations:** Scientific Institute of Public Health (WIV-ISP), Platform Biotechnology and Molecular Biology, rue Juliette Wytsmanstraat 14, 1050 Brussels, Belgium; Service Commun des Laboratoires, 13 chemin du routoir, 67400 Illkirch-Graffenstaden, France

**Keywords:** Identification, Event-specific, GMO, Unauthorized GM-*Bacillus subtilis*, Riboflavin, Vitamin B2, qPCR

## Abstract

**Background:**

Recently, the presence of an unauthorized genetically modified (GM) *Bacillus subtilis* bacterium overproducing vitamin B2 in a feed additive was notified by the Rapid Alert System for Food and Feed (RASFF). This has demonstrated that a contamination by a GM micro-organism (GMM) may occur in feed additives and has confronted for the first time,the enforcement laboratories with this type of RASFF. As no sequence information of this GMM nor any specific detection or identification method was available, Next GenerationSequencing (NGS) was used to generate sequence information. However, NGS data analysis often requires appropriate tools, involving bioinformatics expertise which is not alwayspresent in the average enforcement laboratory. This hampers the use of this technology to rapidly obtain critical sequence information in order to be able to develop a specific qPCRdetection method.

**Methods:**

Data generated by NGS were exploited using a simple BLAST approach. A TaqMan® qPCR method was developed and tested on isolated bacterial strains and on the feed additive directly.

**Results:**

In this study, a very simple strategy based on the common BLAST tools that can be used by any enforcement lab without profound bioinformatics expertise, was successfully used toanalyse the *B. subtilis* data generated by NGS. The results were used to design and assess a new TaqMan® qPCR method, specifically detecting this GM vitamin B2 overproducing bacterium. The method complies with EU critical performance parameters for specificity, sensitivity, PCR efficiency and repeatability. The VitB2-UGM method also could detect the B. subtilis strain in genomic DNA extracted from the feed additive, without prior culturing step.

**Conclusions:**

The proposed method, provides a crucial tool for specifically and rapidly identifying this unauthorized GM bacterium in food and feed additives by enforcement laboratories. Moreover, this work can be seen as a case study to substantiate how the use of NGS data can offer an added value to easily gain access to sequence information needed to develop qPCR methods to detect unknown andunauthorized GMO in food and feed.

**Electronic supplementary material:**

The online version of this article (doi:10.1186/s12896-015-0216-y) contains supplementary material, which is available to authorized users.

## Background

Riboflavin (vitamin B2) is necessary for basic cell metabolism but is not synthetized by higher animals and needs to be ingested. Therefore, the vitamin is commercialized for nutritional use in the fortification of various food and feed products. It is also commonly used as a colorant in food such as ice cream, cheeses, mayonnaises and sauces, and as a medical identification aid, because of its intense yellow color [1–3].

At the present time, fermentation using microbiological processes is widely applied to commercially produce riboflavin [4, 5] and genetically modified (GM) *Bacillus (B.) subtilis* overproducing riboflavin is one of the main organism currently used for riboflavin’s industrial production [2, 4, 6–8]. One common genetic method used to enhance riboflavin production derived from fermentation by GM *B. subtilis*, is to clone the riboflavin biosynthesis operon (*ribG*, *ribB*, *ribA* and *ribH*) from *B. subtilis* under the control of a strong constitutive promoter into (integrative) plasmids (e.g. pUC19, pBR322) [9–11].

In the framework of EU legislation [12, 13], companies wanting to market in the EU a specific additive produced by Genetically Modified Microorganisms (GMMs), like vitamin B2, need to submit an application that will be evaluated by the European Food Safety Authority (EFSA). This will result in a scientific opinion concerning the safety and the efficacy of the product EFSA [14]. According to the EFSA guidance, food and feed additives produced by GMMs intended for human and animal consumption must be pure. This means that both GMMs (either alive or killed) and newly introduced genes should have been removed from the final product [14]. This is especially important for safety evaluation as the amount of recombinant residual host cell DNA as well as the risk of gene transfer need to be assessed. Based on this information, EFSA will be able to deliver its scientific opinion concerning the product, including whether or not the production strain or its recombinant DNA was detected in the final product (for example see [15]).

According to EU legislation [16, 17], labelling is not required for vitamins and additives produced by a GMM because the producer strain (either alive or killed) and its components, including DNA, are no longer present in the commercialized product as the final product should be carefully purified, not containing any residual GMM material. Therefore, unlike for plant GMOs, the companies do not have to provide an event-specific method to trace the GMO in food and feed products on the EU market and there is no control by enforcement laboratories, for the correctness of the labelling. It is assumed that the company bringing the GMM-derived riboflavin to the market has verified the absence of the GMM or its recombinant DNA in the final product, so that only “pure” product can be found on the EU market. However, very recently, it was demonstrated that GMM-contamination in feed additives could occur as a viable GM *-B. subtilis* was found in an imported lot of vitamin B2 feed additive placed on the EU market. This has led in September 2014 to a notification for unauthorized GMO (UGM) in the European Rapid Alert System for Food and Feed (RASFF 2014–1249 [18]).

Confronted with this RASFF, as no official method is available for detecting this GMM in food and feed, two methods previously developed for other purposes have been proposed and used by enforcement laboratories controlling the GMO-content in food and feed at the EU market. The first one allows identifying the presence of *B. subtilis* strains and includes a PCR amplification of the 16S rRNA gene followed by its sequencing [19]. This method is not especially convenient and is more laborious. Indeed, it requires sequencing in addition to the PCR analysis and this is not commonly used by the routine laboratories in GMO analysis. The second method allows detecting the presence of recombinant DNA. It is a real-time PCR method that was used as an internal amplification control targeting the plasmid pUC19, commonly used to construct genetically modified *B. subtilis*. This method was originally developed in duplex with a qPCR method for the detection of pathogenic *Yersinia enterocolitica* in food samples [20]. It targets a standard vector construct (i.e. pUC19), and consequently has a large spectrum of targets commonly used in many GMM constructs. Moreover, traces of such kind of recombinant DNA could be found in mixes containing optimized *Taq* polymerase enzyme used for PCR. Therefore, these 2 methods both provide ambiguous results in the detection of GM*-B. subtilis* overproducing riboflavin. There is a need to develop a faster, specific method targeting this particular GM-strain to be put at the disposition of the enforcement laboratories.

In order to develop such a specific method, the GM*-B. subtilis* overproducing riboflavin was isolated from three different samples of imported vitamin B2 feed additive and collected by the French competent authorities in the framework of the RASFF 2014–1249. Next generation sequencing (NGS) was performed on the genomic DNA extracted from one of the isolates. The sequencing reads were *de novo* assembled and genome annotation was performed on the contig sequences [21]. However, NGS data analysis often requires appropriate tools, involving bioinformatics expertise which is not always present in the average enforcement laboratory. This hampers the use of this technology to easily obtain critical sequence information in order to be able to develop a specific qPCR detection method.

In this study, the DNA sequences previously generated by the NGS approach [21] were used to develop a TaqMan® qPCR method targeting the junction between the *B. subtilis* riboflavin operon and the vector used to construct this GM-strain. Different performance criteria of the developed qPCR method such as specificity, sensitivity, PCR efficiency and repeatability were evaluated according to the GMO guidelines [22, 23]. Finally, the qPCR method was assessed directly on the gDNA extracts from the three different samples of imported vitamin B2 feed additive and collected by the French competent authorities. The method proposed in the present study provides a crucial tool for identifying specifically and rapidly the GM-*B. subtilis* overproducing riboflavin firstly detected in the imported vitamin B2 feed additive. Moreover, it illustrates that NGS data can be used very easily, without specific bioinformatics expertise, in order to obtain crucial information to develop specific TaqMan® qPCR methods, to detect unknown and unauthorized GMO in food and feed.

## Results and Discussion

### Sequence analysis to identify the junction and design of the GM-specific qPCR assay

**Fig. 1:**
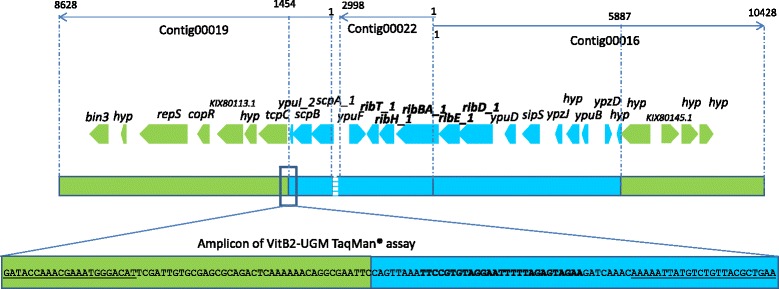
Amplicon, primers and probe sequences of the VitB2-UGM qPCR assay, position of the junction GM-plasmid-insert. Green: similarity with plasmid pSM19035 and pBT233, light blue: “insert” – similarity with *Bacillus subtilis subsp. subtilis str. 168* sequences (including riboflavin biosynthesis genes – in bold); striped blue box: gap of 37 basepairs between Contig00019 and Contig00022, sequence obtained by PCR followed by sequencing; Underlined: primers of the VitB2-UGM qPCR assay, bold: probe of the VitB2-UGM qPCR assay; the size and orientation of the contigs has been indicated at the top, as also the position of the junctions in the respective contigs; Genes located on the respective contigs are indicated with green and light blue arrows using annotation of *B. subtilis* strain 2014–3557, [Genbank: JYFL01000000] – genes encoding following proteins: *bin3* - putative transposon Tn552 DNA-invertase; *hyp* – hypothetical protein; *repS* - protein RepS; *copR* - plasmid copy control protein CopR; KIX80113.1: accumulation-associated protein; *hyp* - hypothetical protein; *tcpC* - conjugative transposon protein TcpC; *ypuI_2* - part of putative protein YpuI_2; *scpB* - segregation and condensation protein B; *scpA-1* - segregation and condensation protein A; *ypuF* - putative protein YpuF; *ribT_1* - protein RibT; *ribH_1* - 6,7-dimethyl-8-ribityllumazine synthase; *ribBA_1* = GTP cyclohydrolase-2; *ribE_1* - riboflavin synthase; *ribD_1*–5-amino-6-(5-phosphoribosylamino)uracil reductase; *ypuD* - putative protein YpuD; *sipS* - signal peptidase I S; *ypzJ* - putative protein YpzJ; *hyp* - hypothetical protein; *ypuB* - putative protein YpuB; *ypzD* - spore germination protein-like protein YpzD; *hyp* - hypothetical protein; *hyp* - hypothetical protein; *KIX80145.1* - N-acetylmuramoyl-L-alanine amidase domain-containing protein; *hyp* - hypothetical protein; *hyp* – hypothetical protein

The first step in the development of the specific qPCR assay consisted of identifying the junction of the GM-insert into the *Bacillus subtilis* GM-strains extracted from vitamin B2 samples 2014–3557 [Genbank: JYFL01000000] [21]. As elaborated in Methods, a BLAST study of the contigs of the previously published sequence of the GM-*Bacillus subtilis* 2014–3557 isolated from vitamin B2 80 % [Genbank:JYFL01000000.1] [21] was used to identify the contigs containing the riboflavin biosynthesis operon *ribGBAH*, with a focus on locating the *ribA* gene as it has been previously reported that additional expression of this gene encoding the rate limiting enzyme in riboflavin synthesis increases riboflavin synthesis even more as compared to only overexpression of *ribGBAH* [7, 11, 24]. The overlapping contigs Contig0022 [Genbank:JYFL01000022.1] and Contig0016 [Genbank:JYFL01000016.1] were retrieved as containing genes having 100 % similarity with *B. subtilis* subsp. *subtilis* str. 168 genes *ribH, ribBA, ribE and ribD*, involved in riboflavin biosynthesis [7, 25], as it could also be deduced from the annotation of the GM-*B. subtilis* genome sequence [Genbank:JYFL01000000] [21] (Fig. 1). Using the publicly available genome sequence of *B. subtilis* subsp. *subtilis* str. 168 [Genbank:CP010052.1] and a BLAST analysis, Contig00019 was identified as the other contig adjacent to Contig00022 (Fig. 1). However, a gap of 37 basepairs between Contig00019 and Contig00022 was observed when comparing these contig sequences to the *B. subtilis* subsp. *subtilis* str. 168 genome sequence [Genbank:CP010052.1] . PCR and sequence analysis was used to confirm that the genomic regions present on both contigs are indeed adjacent in the GM-*Bacillus* genome (see Additional file 1: Figure S1 and Additional file 2: Figure S2). Further investigation of Contig00019 and Contig00016 revealed the presence of a region with 100 % similarity to the pSM19035 [Genbank:AY357120.1] or pBT233 [Genbank:NG_034603.1] plasmid (Fig. 1). It needs to be further investigated whether these plasmid sequences are integrated in the GM-*Bacillus* genome. Nevertheless, this vector has probably been used to construct the GM-strain. Therefore, the junctions between the non-naturally present vector sequences and the *Bacillus* region containing the riboflavin biosynthesis genes (GM-cassette) were to be targeted by the event-specific TaqMan® qPCR assay. The GM-cassette junctions were found around position 1454 in the contig0019 and around position 5887 in the contig0016 (Fig. 1). Primer pairs and probes have been designed at these positions and tested on the GM-*Bacillus subtilis* 2014–3557 (data not shown). One of the assays targeting the junction on the Contig0019 was kept for further evaluation (Table 1 and Fig. 1). This assay, called the VitB2-UGM qPCR assay, was then evaluated for its specificity.

**Table 1:** Primer pair sequences, position on contig 19, concentration and amplicon size for VitB2-UGM qPCR assay

Target	Primer pair name	Primer name	Position on contig 19	Primer sequence, 5'->3'-	Primer/probe concentration	Amplicon	Origin pair size
Contig00019 from Bacillus subtilis GM overproducing riboflavin 2014–3557 [Genbank:JYFL01000019.1]	VitB2-UGM	VitB2-UGM-F	1,399–1,420	GAT ACCAAACGAAAT GGGACAT	250 nM	128 bp	this study
		VitB2-UGM-R	1,468–1,494	TTCAG CGT AACAGACAT AATTTTT	250 nM		this study
		VitB2-UGM-P	1,503–1,526	1 ICCGIGIAGGAAI 1 1 1 IAGAGIAGAA	100 nM		this study

### Determination of VitB2-UGM qPCR assay’s specificity

The specificity test allows testing the inclusivity and exclusivity of the VitB2-UGM qPCR assay. The experimental design involved 3 *Bacillus subtilis* GM-strains overproducing riboflavin that were extracted from three samples of imported vitamin B2 80 %, 51 non-target strains representing 28 species belonging to 19 genera, a CTD (DNA diluent) and an NTC (Table 2). The non-target microorganisms to test the exclusivity were chosen among taxonomically closely related (e.g. other *Bacillus* species) and not closely related (pathogenic or not) bacteria that can be present in the environment and in food and feed matrices [26]. In feed/food additives, normally there should not be any bacteria present, as elaborated above. However, some species also known to be used as GMM were included in the specificity test (e.g. *E. coli*, *B. licheniformis*). The VitB2-UGM qPCR assay amplified 100 % (3/3) of the tested GM-*B. subtilis* strains overproducing riboflavin and none (0/51) of the non-target samples nor the No Template Control (NTC) nor the diluent DNA (CTD) (Table 2). Thus, the detection of the GM-*Bacillus subtilis* 2014–3557 using the VitB2-UGM qPCR assay is 100 % specific for the GM-strain. This was somehow expected as the VitB2 UGM qPCR assay is a construct specific assay (designed on the junction between the between inserted endogenous riboflavin biosynthesis genes (“GM-cassette”) and non-naturally present sequences. The presence of this junction is not expected in wild-type bacteria, no matter what their origin or phylogenetic relationship is. The sequence of the amplicon was determined and corresponded to the expected one (data not shown).

**Table 2:** Selectivity assessment of the VitB2-UGM qPCR assay for detection of the GM-*Bacillus subtilis* 2014–3557 overproducing riboflavin

Bacterial species	Origin	Reference	Cq
Bacillus subtillis GM (2014–3557)	SCL	2014–3557	22.28
Bacillus subtillis GM (2014–3558)	SCL	2014–3558	26.10
Bacillus subtillis GM (2014–3559)	SCL	2014–3559	25.08
Bacillus subtillis subtilis (24 isolates)	UCL	^a^	N/A
Bacillus cereus	IPH-FP	ATCC 14579	N/A
Bacillus lentus	IPH-FP	TIAC 0101	N/A
Bacillus licheniformis	IPH-FP	TIAC 0102	N/A
Bacillus sphaericus	IPH-FP	TIAC 0104	N/A
Bacillus thuringiensis	IPH-FP	TIAC 0096	N/A
Bacillus mycoides	IPH-FP	TIAC 0097	N/A
Bacillus circulans	IPH-FP	TIAC 0100	N/A
Escherichia coli	IPH-FP	ATCC 25922	N/A
Shigella boydii 10	Salm -NRC	12–2582	N/A
Aeromonas hydrophila	IPH-QML	6688 (M/2862)EEQ 2003/2	N/A
E. coli O145:H28	IPH-FP	TIAC 1360	N/A
Campylobacter jejuni	IPH-FP	ATCC 33291	N/A
Citrobacter freundii	IPH-FP	TIAC 0554	N/A
Enterobacter aerogenes	IPH-QML	3778 (M/317-M/3785) EEQ 2002/3	N/A
Streptococcus pyrogenes	IPH-QML	ATCC 19615	N/A
Hafnia alvei	IPH-QML	7186	N/A
Klebsiella pneumoniae	IPH-FP	TIAC 0446	N/A
Listeria monocytogenes	List- NRC	ATCC 51772	N/A
Proteus mirabilis	IPH-FP	TIAC 0726	N/A
Providencia rettgeri	IPH-QML	1521 (M/831) EEQ 1999/2	N/A
Pseudomonas aeruginosa	IPH-FP	LMG 6395	N/A
Salmonella enterica enterica Enteritidis	Salm-NRC	H,VI,6,32	N/A
Salmonella enterica enterica Typhimurium	Salm-NRC	H,II,13,13	N/A
Serratia marcescens	IPH-QML	7015	N/A
Staphylococcus aureus	IPH-FP	ATCC 25923	N/A
Vibrio parahaemolyticus	IPH-FP	TIAC 0610	N/A
Yersinia enterocolitica	IPH-FP	LMG 15558	N/A
NTC			N/A
CTD			N/A

Cq: Cq value obtained with the VitB2-UGM qPCR assay under the conditions described in Methods; N/A: no amplification; ^a^: reference numbers of *B. subtilis* strains used: W04-S10, E07-S05, E08-S06, W05-S03, W10-S01, E11-S02, E12-S04, E14-S06, E16-S07, W13-S04, E23-S03, W16-S03, E28-S04, E33-S01, E34-S01, E38-S02, W24-S03, SI0005, SI0212, SUB033, SUB043, SUB056, BNBs4, BNBs6; SCL: Service Commun des Laboratoires, Illkirch-Graffenstaden, France; UCL: Food and Environmental Microbiology, Earth and Life Institute, Faculty of Bioscience Engineering, Université Catholique de Louvain, Louvain-la-Neuve, Belgium; IPH-FP: Foodborne pathogens service, Scientific Institute of Public Health, Brussels, Belgium; Salm-NRC: Belgian *Salmonella* and *Shigella* National Reference Center, Scientific Institute of Public Health, Brussels, Belgium; IPH-QML: Quality of Medical Laboratories, Scientific Institute of Public Health, Brussels, Belgium; List-NRC: Belgian *Listeria* National Reference Center, Scientific Institute of Public Health, Brussels, Belgium

### Determination of VitB2-UGM qPCR assay’s dynamic range and PCR efficiency

**Fig. 2:**
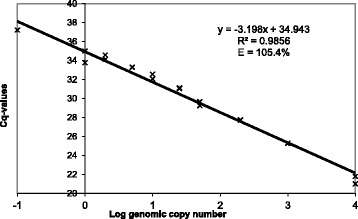
Dynamic range, coefficient of determination (R^2^) and PCR efficiency (E) of the developed VitB2-UGM qPCR assay. Data were obtained from two replicates at each concentration expressed in estimated copy number of genome

The dynamic range of a qPCR assay is the range of concentrations where the assay performs linearly. This was assessed for the VitB2-UGM qPCR assay by the analysis in duplicate of a serial dilution of gDNA (10,000 to 0.01 theoretical genomic copies) of the GM-*Bacillus subtilis* strain 2014*–*3557 overproducing riboflavin [21]. In addition, this analysis allowed for the assessment of the coefficient of determination (R^2^) and the PCR efficiency (E). The coefficient of determination (R^2^) is an indicator of how well the data fit the linear regression curve. The VitB2-UGM qPCR assay performed linearly between 1 and 10,000 genomic copies as its R^2^-value, i.e. 0.99, exceeds the required 0.98 [22, 23] (Fig. 2). From the dynamic range analysis, the PCR efficiency (E) was calculated. The VitB2-UGM qPCR assay displayed a PCR efficiency of 105.4 %, thereby corresponding to the accepted limits fixed for qPCR qualitative method used to detect GMO of 80–120 % as well as to the ones used to quantify GMO of 90–110 % [22, 23].

### Determination of VitB2-UGM qPCR assay sensitivity and repeatability

The sensitivity test was performed to determine the LOD of the VitB2-UGM qPCR assay. The LOD is defined as the lowest concentration of an analyte giving a positive result with a probability of 95 % [22, 23]. The LOD of the VitB2-UGM qPCR assay was determined to be between 1 and 2 copies (Table 3) complying with the requirement “below 25 genomic copies” [23, 27]. The r-values of the Cq-values ranged between 1.3 to 3.2 Cq. The RSDr-values of the Cq-values were between 1.4 to 3.3 % (Table 3). There is no limit fixed for these criteria for qualitative qPCR methods [27].

**Table 3:** Limit of detection determination and repeatability of Cq-values of the VitB2-UGM qPCR assay

Estimated copy number	Mean Cq values ± SD	Positive signals (%)	RSDr	r
10	31.99 ± 0.73	100	2.3	2
5	33.01 ± 0.47	100	1.4	1.3
2	34.86 ± 1.15	100	3.3	3.2
1	35.71 ± 1.07	94.4	na	na
0.1	36.41 ± 0.09	22.2	na	na
DBC (negative control)	N/A	0	na	na

Average, standard deviation and percentage of positive signal of Cq-values and statistical analysis of the Cq values obtained at each dilution point (Total repeats *n*=18). N/A: no amplification; na: not applicable. DBC: dilution buffer control

### Test of the VitB2-UGM qPCR assay on vitamin B2 80 % feed additive samples

To verify if the developed VitB2-UGM qPCR assay targeting the GM-*Bacillus subtilis* 2014–3557 overproducing riboflavin is able to detect this GM strain in real-life samples, the developed qPCR assay has been tested on gDNA extracts from the three samples of imported vitamin B2 80 % feed additive and containing these GM-*Bacillus* strains.

The DNA extracted from each of the samples gave a clear signal when using the VitB2-UGM qPCR assay at a Cq-value around 14 for 10 times dilutions and around 17.5 for 100 times dilutions (Table 4).

**Table 4:** Test of VitB2-UGM qPCR assay on Vitamin B2 80 % samples

Sample	Dilution	Average Cq-value
Sample vitamin B2 80 % (2014–3557)	1/10	13.9
	1/100	17.4
Sample vitamin B2 80 % (2014–3558)	1/10	14.4
	1/100	17.7
Sample vitamin B2 80 % (2014–3559)	1/10	14.3
	1/100	17.6
NTC	na	N/A

*na* not applicable, *N/A* no amplification

This demonstrates that the VitB2-UGM qPCR assay can be used to detect the GM-*Bacillus* strain in real-life samples.

## Conclusions

As no method was available to specifically detect the presence of an unauthorized GMO consisting of a GM *Bacillus subtilis* bacterium overproducing vitamin B2 found in a feed additive (RASFF 2014–1249), in the present study a new TaqMan® qPCR method specifically detecting this GM vitamin B2 overproducing bacterium was designed. To be able to develop the VitB2-UGM qPCR assay, critical sequence information was retrieved and used from massive sequence data previously obtained by an NGS approach [21]. To analyse these data, a very simple strategy based on the common BLAST tools, that can be used by any enforcement lab without profound bioinformatics expertise, was successfully followed. This allowed to design a specific qPCR method targeting the junction between inserted endogenous riboflavin biosynthesis genes (“GM-cassette”) and non-naturally present sequences (vector).

To guarantee the high-standard quality of the VitB2-UGM method, acceptance parameters like specificity, sensitivity, PCR efficiency and repeatability commonly used to validate a qualitative qPCR method for the detection of GMO were evaluated [22, 23, 27]. The method complies with the acceptance criteria for all of the assessed parameters. In addition, to further assess the applicability of the newly developed method not only for isolated strains but also for feed products, the VitB2-UGM method was successfully tested on gDNA immediately extracted from the feed additive from where the GM-strains were isolated. This confirms its effective use in routine analysis for reliable detection by enforcement laboratories of the GM- *B. subtilis* bacterium overproducing vitamin B2 (RASFF 2014–1249). In the future, this specific method can be used to screen feed and food products regarding this specific GM-bacterium overproducing vitamin B2. However, this method might have a broader application, as vitamin B2 is used in a wide range of products present at both the food and feed market [1]. Moreover, this vitamin is not a unique example of additive produced by GMM. Indeed, GMM are used to produce other vitamins, additives and processing agents for the food industry [1]. Companies are responsible for ensuring that their food and feed put at the market are safe, pure, and that it complies with legislation on food and feed additives and regulation on reducing or eliminating human health risks caused by possible contaminants. However, in view of the RASFF demonstrating the presence of an unauthorized GM (UGM) contaminant in feed, the intensive use of GMM to synthetize food and feed additives, as well the possible import of these products in EU, the question is raised if a more systematic survey of the EU market for GMM food and feed contaminants in additives is not necessary.

Hereto, our study underlines that when this NGS strategy is applied on unknown and unauthorized GMM, it delivers a massive amount of information that can be easily used by enforcement laboratories without specific bioinformatics expertise for the development of specific qPCR methods targeting these UGM in the food and feed market.

## Methods

### Bacterial strains

The bacterial strains used in this study are listed in Table 2. A panel of DNA extracted from 51 bacterial strains (24 *Bacillus subtilis* strains, 7 others species of the *Bacillus* genus (*B.)* and 20 strains from 18 other bacterial genera, all obtained from National Reference Centres and Laboratories and C. Nannan from UCL, Belgium) and 3 GM-*B. subtilis* strains has been tested. The 3 GM-*B. subtilis* strains (2014–3557, 2014–3558 and 2014–3559) were isolated from three samples of imported vitamin B2 80 % feed additive powder and analysed by the French GMO-Laboratory “Service commun des Laboratoires” in the framework of the RASFF 2014–1249. No ethical approval was required for this study as no humans, human data or animals were involved.

### Bacterial growth conditions, DNA extraction quantification and sequencing

For the specificity test, DNA of the *Bacillus* strains (including the GM ones) was extracted by boiling as previously described [28]. For the other strains, DNA was extracted using the “Gram-Negative or Gram-Positive Bacteria” protocol of the DNeasy Blood and Tissue Kit (Qiagen Benelux–B.V., KJ Venlo, the Netherlands) from the pellet of 2 ml of overnight cultures of each bacterial strain grown in Brain-Heart Infusion (BHI) broth at the adequate temperature and oxygen condition. For the sensitivity test, the DNA from the GM-*B. subtilis* 2014*–*3557 strain was extracted from the pellet of 84 ml of overnight culture under the conditions specified above, using the Genomic-tip 100/G (Qiagen, Benelux–B.V., KJ Venlo, the Netherlands). All kits were used according to the manufacturer’s recommendations. The DNA concentration was determined using a Nanodrop® 2000 device. The DNA quality was verified via the A260/A280 and A260/A230 ratio and on agarose gel (1 %). Sequencing was performed on a ABI3130xl Genetic Analyzer (Life Technologies, Gent, Belgium) following the manufacturer’s instructions.

### Sequence analysis to identify the contig containing the junction

To identify which contig of the GM-*B. subtilis* 2014–3557 genome sequence [Genbank:JYFL00000000] [21]) contains the natural riboflavin biosynthesis genes (“GM-cassette”), a megablast of each of the 36 contigs was performed. Contigs matching with riboflavin biosynthesis genes were further analysed by megablast to determine the position of the junction between inserted natural riboflavin biosynthesis genes (“GM-cassette”) and the non-naturally present sequence (vector). These sequences were then used to develop the GM-*B. subtilis* 2014–3557 overproducing riboflavin-specific qPCR assay.

### PCR analysis to confirm the order of the contigs Contig00019 and Contig00016

The PCR was performed in a final reaction volume of 25 μl containing 1× DreamTaq Green Master Mix (Thermo Fisher Scientific), 400 nM of forward (Scaf-19-F3-seq, 5′ TTTCGGTACTCAATCAGCTTTTC 3′) and reverse primer (Scaf-22-R-seq, 5′ CAAGGTGCTTCCTCCTTTAAT 3′), and 5 μl DNA template (corresponding to 10^6^ genomic copies of the GM-*B. subtilis* 2014–3557 strain extracted with the Genomic-tip 100/G kit (Qiagen Benelux – B.V., KJ Venlo, the Netherlands). The following protocol was used in a thermal cycler: 2 min at 95 °C, 35 cycles of 30 s at 95 °C, 30 s at 55 °C and 1 min at 72 °C, 10 min at 72 °C. The PCR product (600 bp) was visualized by agarose gel electrophoresis (1 % agar) with ethidium bromide staining. The PCR product was subsequently purified with the Wizard PCR clean-up kit (Promega Benelux B.V., Leiden, the Netherlands). This DNA was used for bidirectional Sanger sequencing (on a ABI3130XL Genetic Analyzer instrument, according to the manufacturer’s instructions), using the primers Scaf-19-F3-seq and Scaf-22-R-seq. The obtained sequences were aligned to the corresponding one of the *B. subtilis* subsp. *subtilis* str. 168 genome sequence [Genbank:CP010052.1] using DNASTAR Lasergene software.

### Development and *in silico* assessment of primer pairs

The qPCR assay developed makes use of an hydrolysis probe, also known as TaqMan® probe. The design of the primers and probe for the TaqMan® qPCR assay to detect the GM-junction was done using the “Primer 3” program [29, 30] with the “product size range” parameter set at “60 to 120 bp” and “primer size” optimal set at “22 bases” and a part of the DNA sequence of contig-19 (described in [Genbank: JYFL01000000], and containing the *B. subtilis* riboflavin biosynthesis genes (“GM-cassette”) and the non-naturally present sequences (vector) as input target sequence. Subsequently, the selectivity of the designed primer pairs were *in silico* tested. This test was performed using the “wprimersearch” software available on wEMBOSS Open Source Software package [31–33]. This software mimics the PCR amplification with the tested primers and using as template the DNA sequences present in a collection of bacterial DNA sequences of non-GM bacterial genomes of 248 strains, representing 122 species belonging to 72 genera, retrieved from the NCBI public database [34]. Only primer pairs that did not yield an *in silico* amplicon using the DNA sequences of this collection of non-GM bacteria strains were retained for the following steps. As a last verification step, a megablast of the amplicon was performed to verify that no similar sequence exists [35–37].

### VitB2-UGM qPCR assay

All qPCR assays were performed on an Applied Biosystems 7300 Real-Time PCR System (Applied Biosystems, Life Technologies, Gent, Belgium) using MicroAmp® Optical 96-Well Reaction Plates closed with MicroAmp® Optical 8-Cap Strips (Applied Biosystems, Life Technologies, Gent, Belgium). The reaction was performed in a final volume of 25 μl containing 5 μl of the appropriate template (10^4^ copies of gDNA of each of the tested strains (Table 2) for the specificity test or a serial dilution of gDNA of GM-*B. subtilis* strain 2014*–*3557 overproducing riboflavin [21] for the sensitivity test), 1X TaqMan® PCR Mastermix (Diagenode, Liège, Belgium), 250 nM of each primer and 100nM of the probe (Table 1, i.e. VitB2-UGM qPCR assay). The estimated bacterial genomic copy number was calculated according to the genome size of each targeted bacteria (information available in the NCBI database; for *Bacillus* strains, the previously reported genome size of *B. subtilis* of 4, 214, 810 bp was used) using the formula previously presented [38]. The following thermal program was applied: A single cycle of DNA polymerase activation for 10 min at 95 °C followed by 45 amplification cycles of 15 sec at 95 °C (denaturing step) and 1 min at 60 °C (annealing-extension step). The fluorescent reporter signal was normalized against the internal reference dye (ROX) signal and the threshold limit setting was performed in automatic mode, according to the ABI Sequence Detection Software version 1.4 (Applied Biosystems, Life Technologies, Gent, Belgium), unless manual adjustment was considered necessary. “No Template” Controls (NTC) using DNase and RNase free water (Acros, Geel, Belgium) and DNA diluent control using calf thymus DNA (CTD, Invitrogen) at 4 ng/μl were included in each assay to assess respectively primer dimer formation and non-specific amplification. Sanger sequencing on a ABI3130xl Genetic Analyzer (Life Technologies, Gent, Belgium) according to the manufacturer’s instructions, was used to confirm the obtained amplicon.

The qPCR assay gives one result, i.e. the quantification cycle (Cq) value which represents the fractional cycle at which the PCR amplification reaches the threshold level for the reaction [39].

### Dynamic range and calculation of the PCR efficiency

The dynamic range was assessed for the VitB2-UGM qPCR assay by the analysis in duplicate of a serial dilution in a carrier DNA background (4 ng/μL Calf Thymus DNA (CTD) (Invitrogen, Life Technologies, Gent, Belgium)) of gDNA (10,000 to 0.01 theoretical genomic copies) of the GM-*Bacillus subtilis* strain 2014*–*3557 overproducing riboflavin [21]. The carrier DNA avoids the improper dilution due to low concentration of gDNA. The PCR efficiency was calculated according to the formula previously reported [40].

### Sensitivity test and repeatability calculation

The GM-*B. subtilis* strain 2014*–*3557 overproducing riboflavin [21] was used as target. To determine the LOD, a range between 10 and 0.1 theoretical genomic copies was tested (i.e. 10, 5, 2, 1 and 0.1). Each dilution was tested in six replicates. Moreover, the analysis was performed three times independently, under repeatable conditions, resulting in 18 repeats for each dilution point. In order to verify that the DNA concentration used to calculate the LOD was not overestimated, the number of positive signals was recorded over the 18 replicates. The “0.1 copy” was included to verify that the concentration of DNA used to calculate the LOD was approximately correct.

The repeatability of the assay can be evaluated thanks to the independent tests performed, i.e. with the same protocol, with the same samples, by the same operator using the same apparatus within a short interval of time [41]. The repeatability limit (r) and the relative standard deviation of repeatability (RSD_r_) were calculated according to ISO 16140 [42]. The RSD_r_ and r-values of the Cq-values were calculated at each dilution point.

### Extraction of genomic DNA from vitamin B2 samples

Genomic DNA (gDNA) was extracted from feed additive samples using a CTAB-based method adapted from Dellaporta et al. [43]. Four volumes of CTAB extraction buffer (NaCl 1.4 M, EDTA 0.02 M, Tris–HCl 0.1 M, CTAB 2 %), supplemented with Ribonuclase A (at a final concentration of 0.1 mg/ml) were added to 250 mg of vitamin B2 powder, mixed regularly and incubated for 30 min at 65 °C. Next, Proteinase K (at a final concentration of 0.4 mg/ml) was added, mixed regularly and incubated for 30 min at 65 °C. After centrifugation (10 min at 12,000 *g*), the supernatant was collected and 1 volume of chloroform was added and vortexed for 30 sec. After mixing and centrifugation (15 min at 12,000 *g*), the upper phase was collected. 0.6 volumes of isopropanol and 0.4 volumes of ammonium acetate 10 M were added, gently mixed by inversion, incubated for 30 min on ice and centrifuged (15 min at 12,000 *g*). The supernatant was discarded and the pellet was washed with 1 ml of 70 % ethanol and subsequently centrifuged (15 min at 12,000 *g*). The pellet was dried for a few minutes at 37 °C. Finally, the pellet was re-suspended in 350 μl of TE (10 mM Tris-0.2 mM EDTA pH 8.0) pre-warmed at 50 °C for 2 min. gDNA extracts were then stored at +4 °C. For the qPCR analysis, 1/10 and 1/100 dilutions were made in TE (10 mM Tris-0.2 mM EDTA pH 8.0) and analysed in duplicate with the VitB2-UGM qPCR assay as described above.

## Availability of supporting data

The data sets supporting the results of this article are included within the article and its additional files:

Additional file 1: Figure S1: Sequence of B. *subtilis* subsp. *subtilis* str. 168 genome [Genbank:CP010052.1] corresponding to the region containing part of Contig00019 and Contig00022, and the “37 bp gap”.

Additional file 2: Figure S2: Alignment of sequences of PCR fragment containing the “37 bp gap” between Contig00019 and Contig00022 and the B. *subtilis* subsp. *subtilis* str. 168 genome sequence [Genbank:CP010052.1].

## Additional files

Additional file 1: Figure S1. Sequence of *B. subtilis* subsp. *subtilis* str. 168 genome sequence [Genbank:CP010052.1] corresponding to the region containing part of Contig00019 and Contig00022. There is a gap of 37 basepairs (uncoloured region in the figure) between Contig00019 (pink region in figure) and Contig00022 (yellow region in figure), when aligning the obtained contigs to *B. subtilis* subsp. *subtilis* str. 168 genome sequence [Genbank:CP010052.1]. PCR and sequence analysis were used to confirm that the genomic regions present on both contigs are indeed adjacent in the GM-*Bacillus* genome. Hereto, primers Scaf-19-F3-seq (positioned on Contig00019, indicated in green) and Scaf-22-R-seq (positioned on Contig00022, indicated in blue) were used to amplify the flanking regions of the junction between Contig00019 and Contig00022. Subsequently, the obtained PCR fragment was sequenced. (PDF 101 kb) Additional file 2: Figure S2. Alignment of sequences of PCR fragment containing the “37 bp gap” between Contig00019 and Contig00022 and the *B. subtilis* subsp. *subtilis* str. 168 genome sequence [Genbank:CP010052.1]. Alignment of the obtained sequences (“57-scaf19-F3” and “57-scaf22-R”) of the PCR fragment containing the junction between Contig00019 and Contig00022 to the corresponding region in the *B. subtilis* subsp. *subtilis* str. 168 genome sequence [Genbank:CP010052.1] (region taken as displayed in Additional file 1: Figure S1). The nucleotides at the beginning of the obtained sequence are to be ignored, as these are of lower quality, which is known for Sanger sequencing with BigDye Terminator v3.1. The position corresponding to the “37 bp gap” has been indicated with a red box. It is clear that both contigs are adjacent in the GM-*Bacillus* genome, and that the sequence region corresponding to the “gap” was not present in the reads/contigs obtained through the the NGS analysis. (PDF 534 kb)

## Abbreviations

GM: Genetically modified
GMM: Genetically modified micro-organism
RASFF: Rapid alert system for food and feed
NGS: Next generation sequencing
GMO: Genetically modified organism
EU: European union
EFSA: European food safety authority
NTC: No template control
E: PCR efficiency
ENGL: European network of GMO laboratories
UGM: Unauthorized genetically modified organism
BHI: Brain-heart infusion
CTD: Calf Thymus DNA
R^2^: Coefficient of determination
