# Supplementary material for: Use of next generation sequencing data to develop a qPCR method for specific detection of EU-unauthorized genetically modified Bacillus subtilis overproducing riboflavin
Source: BMC Biotechnol. 2015 Nov 11;15:103. doi: 10.1186/s12896-015-0216-y (PMC4642757; doi:10.1186/s12896-015-0216-y)
Supplement: Additional file 1: Figure S1. — Sequence of B. subtilis subsp. subtilis str. 168 genome sequence [Genbank:CP010052.1] corresponding to the region containing part of Contig00019 and Contig00022. There is a gap of 37 basepairs (uncoloured region in the figure) between Contig00019 (pink region in figure) and Contig00022 (yellow region in figure), when aligning the obtained contigs to B. subtilis subsp. subtilis str. 168 genome sequence [Genbank:CP010052.1]. PCR and sequence analysis were used to confirm that the genomic regions present on both contigs are indeed adjacent in the GM-Bacillus genome. Hereto, primers Scaf-19-F3-seq (positioned on Contig00019, indicated in green) and Scaf-22-R-seq (positioned on Contig00022, indicated in blue) were used to amplify the flanking regions of the junction between Contig00019 and Contig00022. Subsequently, the obtained PCR fragment was sequenced. (PDF 101 kb) [file 12896_2015_216_MOESM1_ESM.pdf]

…TCGTTCCTCTAGATTTCAAGCTGTGCACGATTTCGTTCATTCTGGCTTCGATTGGTATGTCCTGTCTTGTAATTGTTG
TTTCCATCGGCCTGTTTATTTTTTTGCGTTTCAGCACTTTTTGAAAAGCTCCGATCATATCATAGACCGTGACAGAGAG
CTTTTGCTCAGACTGTTTTACTTCTTTTGCATATTCACTCAAATCACTCGGCGGTTTCGTGAACGATTTTTGTCTTTCT
TCTTCCCGTTCTTTTAAATCCTTCGCCGCATCTTTATATTTTCGGTACTCAATCAGCTTTTCAATCAGTTCCTCCCGCG
GATCTTCTTCTTCAAGTAATTCATCTTCAAAAAGCTCCTCCTCTTGCTTCGGGAGCAGCATTCTGCTTTTAATGCTGAG
CAGCGTGGCAGCCATAACCAAATACTCGCTGGCAATGTCGAGCTCAAGCACGCGCATCGTATGTACATATAATAAATAT
TGTTCAGTGATCTTCGCCACAGGTATATCATATATGTCAATTTCAAGACGATTGATTAAATGAAGCAGCAGGTCCAATG
GGCCCTCAAACGTATCAATTTTCACTTGATATTCTTCCATCATCTCACCATTTTCAAGTATCACTCTCATTGCCGGAAA
AACTGGCTACCCTATAGTATAAGCCACCAAAAAGCTCTCGTCCAATACTATTTCATATCCTGTTTTGAAGGAGTTTGTA
CAGAATATTCAAAACACAAGAGGTGTTTGCTCGGAGCGCAGAAGCGGGAGAAAAAGCCTTTGCTTCTGGTTTTCCTTTC
TAAGAAGCACTAGAGGCCGGGCGTTCTTTTTTCCTTTTATCCCTTCTATGGTACACTAAAAGCACATTAAAGGAGGAAG
CACCTTGTATCCGAAAGCTTATATAGATTATCTCGTTGAGTTTCATGCGACACGGGATTATTTTGAATGCCATGAAATA
TTAGAAGAATATTGGAAGGAAGATCCGCCAAAGAAACGAAAACGCTATTGGGTGGGCTTTATTCAGCTTGCAGTGGCTT
TATATCATCACAGAAGGCAGAATACAGCCGGGGCAAAAAGACTGATGGCGAACAGCATCC…

Supplementary figure 1: Sequence of *B. subtilis* subsp. *subtilis* str. 168 genome sequence [Genbank:CP010052.1] corresponding to the region containing part of Contig00019 and Contig00022

There is a gap of 37 basepairs (uncoloured region in the figure) between Contig00019 (pink region in figure) and Contig00022 (yellow region in figure), when aligning the obtained contigs to *B. subtilis* subsp. *subtilis* str. 168 genome sequence [Genbank:CP010052.1]. PCR and sequence analysis were used to confirm that the genomic regions present on both contigs are indeed adjacent in the GM-*Bacillus* genome. Hereto, primers Scaf-19-F3-seq (positioned on Contig00019, indicated in green) and Scaf-22-R-seq (positioned on Contig00022, indicated in blue) were used to amplify the flanking regions of the junction between Contig00019 and Contig00022. Subsequently, the obtained PCR fragment was sequenced.
