# Supplementary material for: Use of next generation sequencing data to develop a qPCR method for specific detection of EU-unauthorized genetically modified Bacillus subtilis overproducing riboflavin
Source: BMC Biotechnol. 2015 Nov 11;15:103. doi: 10.1186/s12896-015-0216-y (PMC4642757; doi:10.1186/s12896-015-0216-y)
Supplement: Additional file 2: Figure S2. — Alignment of sequences of PCR fragment containing the “37 bp gap” between Contig00019 and Contig00022 and the B. subtilis subsp. subtilis str. 168 genome sequence [Genbank:CP010052.1]. Alignment of the obtained sequences (“57-scaf19-F3” and “57-scaf22-R”) of the PCR fragment containing the junction between Contig00019 and Contig00022 to the corresponding region in the B. subtilis subsp. subtilis str. 168 genome sequence [Genbank:CP010052.1] (region taken as displayed in Additional file 1: Figure S1). The nucleotides at the beginning of the obtained sequence are to be ignored, as these are of lower quality, which is known for Sanger sequencing with BigDye Terminator v3.1. The position corresponding to the “37 bp gap” has been indicated with a red box. It is clear that both contigs are adjacent in the GM-Bacillus genome, and that the sequence region corresponding to the “gap” was not present in the reads/contigs obtained through the the NGS analysis. (PDF 534 kb) [file 12896_2015_216_MOESM2_ESM.pdf]

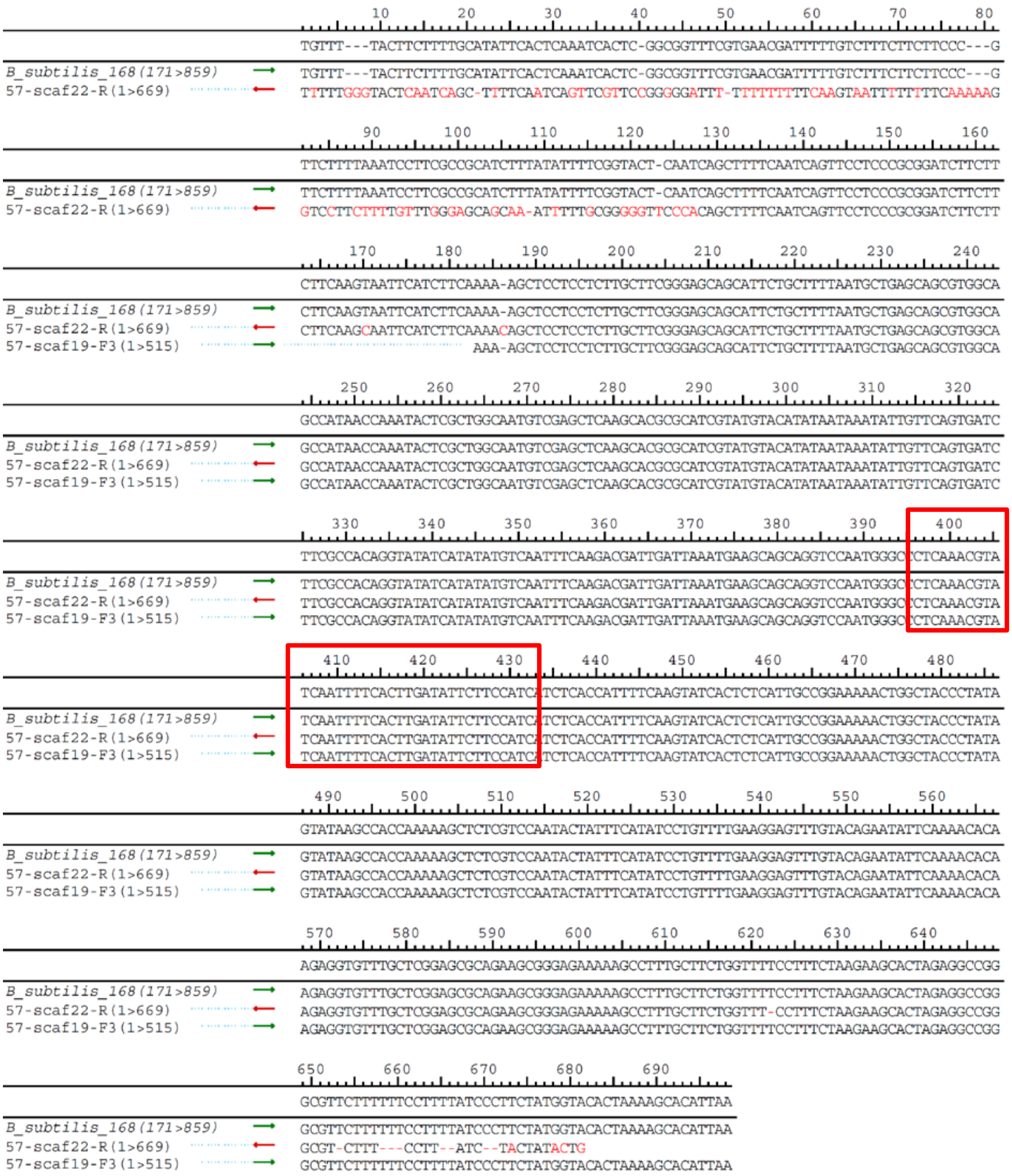

Supplementary Figure 2: Alignment of sequences of PCR fragment containing the "37 bp gap" between Contig00019 and Contig00022 and the *B. subtilis* subsp. *subtilis* str. 168 genome sequence [Genbank:CP010052.1]
Alignment of the obtained sequences ("57-scaf19-F3" and "57-scaf22-R") of the PCR fragment containing the junction between Contig00019 and Contig00022 to the corresponding region in the *B. subtilis* subsp. *subtilis* str. 168 genome sequence [Genbank:CP010052.1] (region taken as displayed in Additional file 1: Figure S1). The nucleotides at the beginning of the obtained sequence are to be ignored, as these are of lower quality, which is known for Sanger sequencing with BigDye Terminator v3.1. The position corresponding to the "37 bp gap" has been indicated with a red box. It is clear that both contigs are adjacent in the GM-*Bacillus* genome, and that the sequence region corresponding to the "gap" was not present in the reads/contigs obtained through the the NGS analysis.
